# Therapeutic potential of mesenchymal stem cells for peripheral artery disease in a rat model of hindlimb ischemia 

**DOI:** 10.22038/ijbms.2021.55861.12491

**Published:** 2021-06

**Authors:** Amani M. El Amin Ali, Amira S. Ahmed, Dina F. El-Yasergy, Moustafa A. Abousarie, Ramadan M. Elsayed, Yasmin E. Mohammed, Rahab A. Mohammed

**Affiliations:** 1 Department of Medical Physiology, Faculty of Medicine, Fayoum University, Fayoum, Egypt; 2 Hormones Department, Medical Research Division, National Research Centre, Giza, Egypt; 3 Department of Pathology, Faculty of Medicine, Cairo University, Cairo, Egypt; 4 Department of Pathology, Faculty of Medicine, Fayoum University, Fayoum, Egypt; 5 Department of Medical Anatomy, Faculty of Medicine, Fayoum University, Fayoum, Egypt

**Keywords:** CXC chemokine receptor 4, Mesenchymal stem cells, Peripheral vascular disease, Vascular endothelial growth - factor receptor 2, von Willebrand factor

## Abstract

**Objective(s):**

Mesenchymal stem cells are viewed as the first choice in regenerative medicine. This study aimed to elucidate the influence of BM-MSCs transplantation on angiogenesis in a rat model of unilateral peripheral vascular disease.

**Materials and Methods:**

Twenty-one rats were arbitrarily allocated into three groups (7/group). Group I: control sham-operated rats, Group II: control ischemic group: Rats were subjected to unilateral surgical ligation of the femoral artery, and Group III: ischemia group: Rats were induced as in group II, 24 hr after ligation, they were intramuscularly injected with BM-MSCs. After scarification, gastrocnemius muscle gene expression of stromal cell-derived factor-1 (SDF-1), CXC chemokine receptor 4 (CXCR4), vascular endothelial growth factor receptor 2 (VEGFR2), von Willebrand factor (vWF), and hypoxia-inducible factor-1α (HIF-1α) were analyzed by quantitative real-time PCR. Muscle regeneration and angiogenesis evaluation was assessed through H&E staining of the tissue. Furthermore, Pax3 and Pax7 nuclear expression was immunohistochemically assessed.

**Results:**

Rats treated with BM-MSCs showed significantly raised gene expression levels of SDF-1, CXCR4, VEGFR2, and vWF compared with control and ischemia groups. H&E staining of the gastrocnemius showed prominent new vessel formation. Granulation tissue within muscles of the ischemic treated group by BM-MSCs showed cells demonstrating nuclear expression of Pax3 and Pax7.

**Conclusion:**

BM-MSCs transplantation has an ameliorating effect on muscle ischemia through promoting angiogenesis, detected by normal muscle architecture restoration and new blood vessel formations observed by H&E, confirmed by increased gene expression levels of SDF-1, CXCR4, VEGFR2, and vWF, decreased HIF-1α gene expression, and increased myogenic Pax7 gene expression.

## Introduction

Peripheral artery disease (PAD) is a mutual vascular complication associated with significant morbidity with a prevalence of more than 20% in individuals aged 80 years or older (1). It is chiefly triggered by atherosclerosis of lower leg arteries, resulting in reduction in blood flow to the muscles of the legs. The chief risk factors include smoking, disturbance in lipid profile, diabetes mellitus, and increase in blood pressure (2). Patients with PAD presented intermittent claudication, leg discomfort, or acute or chronic limb ischemia. Associated signs may include decreased pulse, decreased capillary refill, and trophic changes (3). Severe cases can be presented by ulceration or gangrene of the foot with subsequent increasing risks of amputation and/or death (4). Bone marrow mesenchymal stem cells (BM-MSCs) are a promising source for tissue regeneration with different clinical applications (5). MSCs are widely distributed cells that preserve the ability for postnatal auto-renewal and multilineage differentiation. They have the capacity to secrete anti-inflammatory and anti-fibrotic factors and to stimulate precursors resident in the tissue. In cardiovascular medicine, many studies showed promising results after administration of MSCs in patients having either ischemic or non-ischemic cardiomyopathy by stimulating angiogenesis, regeneration of cardiac muscle cells, and reducing fibrosis (6). Angiogenesis is the formation of new blood vessels from the present vasculature occurring in several organs including skeletal muscles under different physiological and pathological conditions (5) and plays a crucial promising therapeutic method for PAD. Many animal studies have confirmed the role of MSCs transplantation in improving PVD (7). 

The MSCs can facilitate new blood vessel formation through differentiation, cell contact interaction, or paracrine effects (8). Angiogenic mediators generated by MSCs involving vascular endothelial growth factor/vascular endothelial growth factor receptor 2 (VEGF/VEGFR2), transforming growth factor-β (TGF-β), and stromal cell-derived factor-1 (SDF-1) facilitate tissue regeneration (9). VEGFR-2 serves as a positive signal transducer in vascular endothelial cell development and differentiation (10). SDF-1 is a chemokine that can adjust numerous normal processes, such as stimulating the proliferation of endothelial cells (ECs) and development of capillary tubes (11). SDF-1 and its receptor, CXC chemokine receptor 4 (CXCR4), can promote local angiogenesis (12). Additionally, an *in vivo* investigation (13) revealed that engrafted MSCs were positive for von Willebrand factor (vWF), proposing their differentiation into ECs. However, hypoxia-inducible factor-1α (HIF-1α) is a transcription factor that responds to declines in available oxygen (hypoxia) (14). The signaling cascade of HIF mediates the hypoxia effects. Hypoxia retains cells from differentiation and stimulates blood vessels formation (15). The inactivity nature of stem cells is elucidated by its expression of HIF-1α. Whereas, stem cells metabolically remain at a low rate to preserve the stem cells’ potency for long periods in an organism’s life cycle (16).

During regeneration of muscles from stem cells, myogenic progenitor cells are 80–90% positive for Pax3 and 20–30 % positive for Pax7 within 10 days, followed by positivity for MyoD and desmin from day 10 to day 18, followed finally by myogenin. Pax3 and Pax7 are transcription factors and crucial regulators of myogenic cell differentiation. Both of them are early expressed in myogenic progenitor cells, playing a regulatory role in myogenesis (17).

The increased incidence of ischemia with high rates of mortality, complex pathogenesis, and general difficulties in treatment made ischemia a challenge for scientists and clinicians. Therefore, the goal of the current research was to assess the impact of BM-MSCs transplantation on angiogenesis in a rat model of unilateral peripheral vascular disease (unilateral femoral artery ligation).

## Materials and Methods

***Animals***

In this work, twenty-one adult male ordinary strain albino rats, weighing between 150 and 200 g each, were obtained from the animal center in Fayoum University. All rats were placed in clean suitably ventilated cages and were fed the regular laboratory diet with adequate water supply and allowed to acclimatize for 3–4 days on a 12:12-hr light-dark cycle in the laboratory of the medical physiology department, Faculty of Medicine, Fayoum University, Egypt. The local Animal Care Committee of Fayoum University approved the experimental protocol (approval number protocol; N: R201). The experimental procedures were executed in accordance with International Guidelines for Care and Use of Laboratory Animals. The animals were equally distributed into three main groups.

***Experimental design***

**Group I** (control sham-operated rats) (n = 7): Rats were subjected to sham surgery and served as control I group. 

**Group II** (control ischemic group) (n=7): Ischemia was induced in these animals by surgical ligation of the femoral artery at the femoral triangle in one limb with no further intervention and served as control - II group. 

**Group III** (ischemic treated group) (n= 7): Ischemia was induced in the rats by the same procedure as group II and after 24 hr, the rats were injected intramuscularly with a single dose of 5× 10^6^ BM-MSCs) (18).

Two weeks following the end of the experiment, the animals were sacrificed. The muscle tissues (the gastrocnemius muscle) were rapidly dissected out, washed immediately with saline, and processed for gene expression analysis using quantitative real-time PCR, histopathological evaluation, and immunohistochemistry.

***Ligation of femoral artery***

The animal was placed in a supine position. The hair of the left hindlimb was carefully shaved and the whole limb was sterilized. Following anesthesia induction with ketamine (100 mg/kg), a ventral longitudinal midline incision was performed in the left hindlimb and the left femoral artery was freely dissected, separated from the tissues around it, and ligated by a nylon suture. Tissues were kept moist with sterile saline throughout the entire procedure. The skin incision was closed with sterile silk sutures, the wound was sterilized, and postoperative analgesics and antibiotics were used to relieve the pain and prevent the infection. Terramycin and Voltaren were injected and a skin ointment (Garamycin) was used to prevent sepsis. The rats were allowed to recover with free access to food and water for 4 weeks. (19). Sham-operated animals (control group) underwent identical surgical treatment, including isolation of left femoral artery; however, artery ligation was not performed. Ischemia was determined by pale cold limb with trophic changes as ulcers in the left lower limb and loss of the subcutaneous fat; and toe necrosis was seen in almost all of the animals of the ischemic group while these changes were absent in the treated group.

***Isolation, preparation, and identification of bone marrow mesenchymal stem cells (BM-MSCs)***

Briefly, the bone marrow of the femurs and tibias of rats was flushed with phosphate-buffered saline (PBS). By addition of 15 ml Ficoll-Paque (Gibco-Invitrogen, Grand Island, NY, USA), the layer of flushed bone marrow, after being layered and centrifuged, was removed while the layer of nucleated cells was isolated, washed twice in PBS, and centrifuged. The obtained BM-MSCs were layered and supplemented with 10% fetal bovine serum (FBS) [heat inactivated, qualified, One Shot™, United States, Gibco™], 0.5% penicillin/ streptomycin, and incubated at 37 °C and 5% CO_2_ until reaching 80–90% confluence. After 7 days, they were collected with 0.25% trypsin-EDTA (Gibco, BRL, USA) and resuspended in other flasks. After washing and centrifugation at 1800 RPM for 10 min, cell pellets were resuspended with serum-supplemented medium, cultures were incubated at 37 °C in a 50 cm^2^ culture flask (GIBCO/BRL) with 5% CO_2_ environment and saturated humidity. First passage cultures of BM-MSCs were used in the experiment. BM-MSCs were identified by their spindle-shaped morphology. More characterization of BM-MSCs was achieved by flowcytometry (FACS) (Beckman Coulter). The BM-MSCs were suspended (1x 106 cells/ml) and stained with FITC conjugated monoclonal antibodies, CD133, CD34, and KDR (Biolegend) (20).

***Real-time quantitative polymerase chain reaction***

After animal scarification, quantitative RT-PCR gene expression was assessed and histopathological examination of gastrocnemius of the studied rats was done.

Gene expressions of SDF-1, CXCR4, vascular endothelial growth factor receptor 2 (VEGFR2), vWF, and HIF-1α were analyzed by quantitative real-time PCR (*qRT-PCR)*. Total RNA was extracted from homogenates of muscle tissue samples using RNeasy Purification Reagent (Qiagen, Valencia, CA, USA). RNA concentrations and purity were measured with a UV spectrophotometer. Reverse transcription was carried out with 2 μg of total RNA and Superscript III reverse transcriptase (Fermentas, Waltham, MA, USA) for the production of cDNA. qRT-PCR was carried out using SYBR Green PCR Master Mix (Applied Biosystems, Foster City, CA, USA). The primers used for real-time PCR are presented in Table 1. The amplified cDNA by PCR was executed under the following thermal conditions: 50 °C for 2 min (1 cycle), 95 °C for 15 sec, 60°C for 30 sec, and 72°C for 30 sec (40 cycles) which were followed by 60 °C for 10 min (1 cycle). Relative expressions of the threshold cycle (Ct) and the fold-changes (FC) of the studied genes were determined using the equation 2^-ΔΔCt ^(21). GAPDH gene expression as a housekeeping gene was used as internal control.

***Histopathological evaluation***

Muscle tissues from the three groups were dissected and then fixed in 10% buffered formalin, processed, and embedded in paraffin. Each paraffin block was re-cut at 4–5 micron thickness by a rotatory microtome then mounted on glass slides and stained by H&E for histopathological examination and on charged slides for immunostaining of Pax3 and Pax7.

***Immunohistochemistry***

*Pax3 immunostaining*

The slides were put in a Dako autostainer link 48, using a polymer-based detection system (DakoEnVisionTM FLEX, K8000) in which these steps were sequentially performed: incubation for 5 min in 3% H_2_O_2_ to avoid endogenous peroxidase activity, washing the slides by PBS at pH 7.2–7.4., then they were placed in citrate buffer then heated in a microwave oven at 100 °C for 3 successive times, five minutes each for antigen retrieval. The slides were then incubated with polyclonal rabbit anti-mouse/human Pax3 (catalog number # 38-1801, manufactured by Invitrogen/Thermo Fisher Scientific, MA, USA). It was used at 1/100 dilution and incubated for 30 min at room temperature. The slides were washed with PBS at pH 7.2–7.4, followed by application of the Envision Dako link kit optimized for Dako cytomation automated system for 30 min. The slides were washed with PBS at pH 7.2–7.4. 3,3’-di-amino-benzidinetetrahydrochloride (DAB) was applied as chromogen for 5 min. Then for 5 min, the slides were rinsed in distilled water. The slides were removed from the autostainer and Mayer’s Hematoxylin counterstain was done. Dehydration of the slides by ascending grades of alcohol and clearing in xylene for 3 changes was performed and coverslips were applied. Sections were examined by the two researchers and assessed either as positive or negative nuclear staining.

*Pax7 immunostaining*

Used the same steps, but the slides were incubated with mouse monoclonal Pax7 antibody, 1:100 dilution (catalog number # sc-81648, manufactured by Santa Cruz Biotechnology, USA). Sections were examined by the two researchers and assessed either as positive or negative nuclear staining. Six sections from each sample were used for the average positive or negative immunoactivity.

***Statistical analyses***

The Statistical Package for the Social Sciences (SPSS; V. 21.0, Inc., USA) was used to conduct statistical analysis of the data. The presentation of the data took mean ± SD form. Unpaired t-test with Tukey correction was executed for ANOVA-based and *post hoc* pairwise comparisons of the different experimental groups. Moreover, Pearson’s correlation (r) was used to appraise how the various biochemical parameters were correlated with one another. While VEGFR2, vWF, CXCR4, and SDF-1 were assessed using linear regression analysis. The chosen independent variables were VEGFR2, vWF, and CXCR4. Statistical significance was indicated by a *P*-value≤0.05.

## Results

***MSCs isolation, culture, and identification of MSCs***

**Figure 1:**
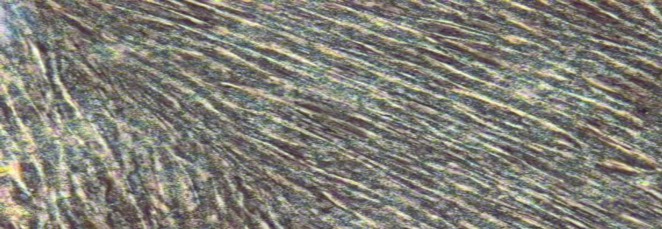
Morphological spindle shape of mesenchymal stem cells (MSCs) identified after 14 days of incubation

MSCs were identified by their morphological spindle shape as presented in Figure 1.

***Phenotypic identification MSCs***

**Figure 2:**
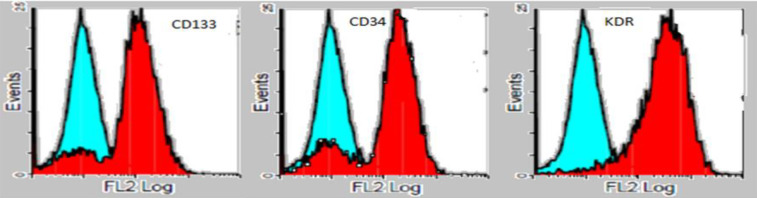
Phenotypic identification of mesenchymal stem cells (MSCs)

MSCs were positive for CD133, CD34, and KDR as illustrated in Figure 2.

***Effect of bone marrow mesenchymal stem cells (BM-MSCs) treatment on the gene expression levels of CXCR4, SDF-1, HIF-1α, VEGFR2, and vWF***

**Figure 3:**
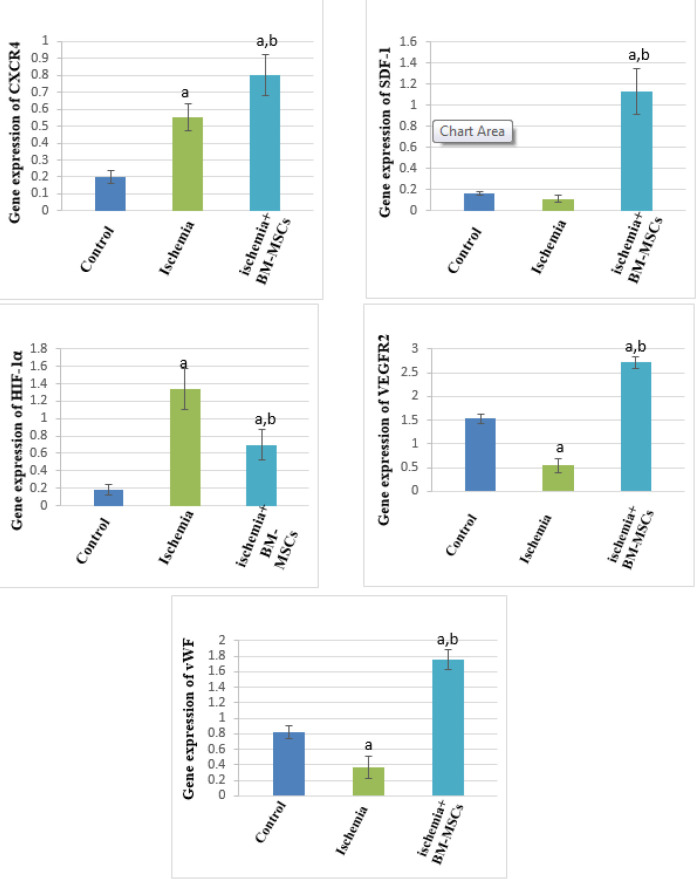
Effect of bone marrow mesenchymal stem cells (BM-MSCs) treatment on the gene expression levels of CXCR4, SDF1, HIF-1α, VEGFR2, and vWF (7 rats/group). Values are expressed as means ± SD. CXCR4: C-X-C chemokine receptor 4. SDF-1: Stromal cell-derived factor-1. HIF-1α: Hypoxia-inducible factor-1 α. VEGFR2: Vascular endothelial growth factor receptor 2. vWF: von Willebrand factor. a Significant difference from the control group. b Significant difference from the ischemia group. *P*-values≤0.05 considered significant

Under optimization of PCR conditions, amplification specificity and efficiency of all gene expressions of RT-qPCR array were verified. Figure 3 reveals the change in the expression levels of CXCR4, SDF1, HIF-1α, VEGFR2, and vWF in the different investigated groups. The relative quantity analysis of real time-qPCR showed an elevation of CXCR4 gene expression by 0.548 and 0.8-fold with ischemia and BM-MSCs treatment, respectively. A significant increase in the gene expression level of CXCR4 in the rats with ischemia or treated with stem cells relative to that in the control group was recorded. However, post-treatment of rats with BM-MSCs significantly raised the gene expression level of CXCR4 compared with ischemia. Regarding SDF-1, its gene expression was found to be high in the rats treated with BM-MSCs by 1.126-fold. In comparison with control and ischemia groups, its expression significantly increased by 0.97 and 1.014-fold, respectively. Additionally, a high expression of the HIF-1α gene was monitored with ischemia (1.336-fold) and post-treatment of rats with BM-MSCs (0.703-fold). A significantly high level of HIF-1α gene expression was registered in the ischemia group and rats treated with BM-MSCs compared with the control group. Whereas, it showed a significant reduction in gene expression with BM-MSCs treatment compared with the ischemia group. A significant decrease in VEGFR2 and vWF expression levels was observed in the ischemia group compared with the control group. Conversely, gene expression levels of VEGFR2 and vWF significantly increased in rats treated with BM-MSCs relative to the control and ischemia groups. The previous results confirm increased angiogenic factors that induce angiogenesis.

***Correlations between different investigated biomarkers***

Correlations between different investigated markers in ischemia and BM-MSCs groups are shown in Table 2. Expression of VEGFR2 exhibited significant positive correlation with gene expression of vWF, CXCR4, and SDF-1 (r=0.984, 0.828 and 0.941, *P*<0.01, respectively). Furthermore, vWF expression was significantly positively correlated with gene expression of CXCR4 (r=0.755, *P*<0.01) and SDF-1 (r=0.942, *P*<0.01). The current results revealed significant positive correlation between gene expression of CXCR4 and SDF-1 (r=0.739, *P*<0.01). 

***Histopathological examination***

**Figure 4:**
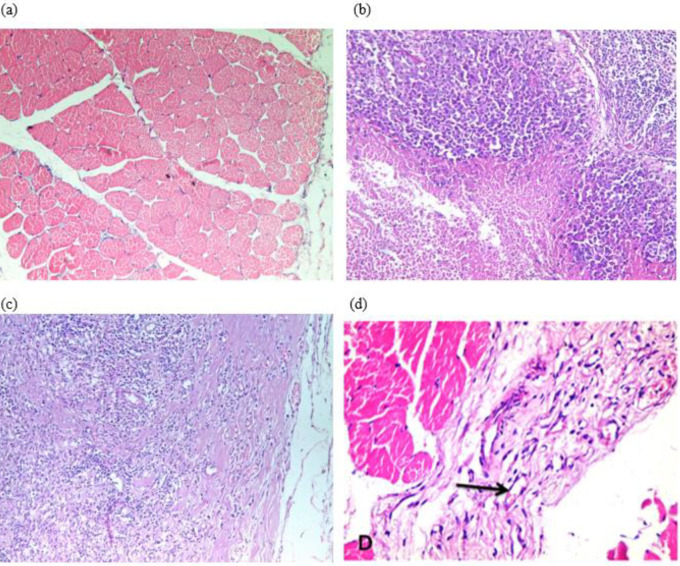
Histological section of the gastrocnemius muscle stained with Hematoxylin and Eosin stain. (a) Normal muscle from the control group. (b) Muscle from the ischemic non-treated group showing necrosis with dense inflammatory reaction and necrosis (c) Muscle from the ischemic treated group by BM-MSCs showing granulation tissue with mild inflammatory reaction and areas of fibrosis (Hematoxylin and Eosin, original magnification, x200). (d) An arrow showed loose connective tissue with many new vessels (HE X 400) in muscle treated by BM-MSCs

The muscles of the control group (7 rats) showed unremarkable pathological changes (Figure 4a). The muscles of the ischemic non-treated group (7 rats) and the treated ischemic group by BM-MSCs showed a necro-inflammatory reaction, granulation tissue, and focal areas of fibrosis (Figures 4b and 4c). Loose connective tissue with many new vessels (HE X 400) in muscle treated by BM-MSCs was observed (Figure 4d).

***Effect of bone marrow mesenchymal stem cells (BM-MSCs) treatment on Pax3 and Pax7 nuclear expression***

**Figure 5:**
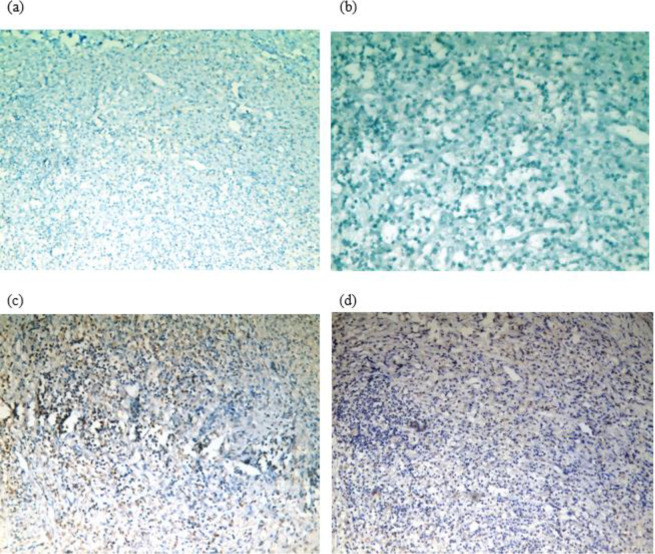
Photomicrograph of muscle sections in ischemic non-treated and ischemic treated groups by BM-MSCs stained for Pax3 and Pax7 nuclear expression. (a) The granulation tissue of the ischemic non-treated group showing negative expression of Pax3 (diaminobenzidine, original magnification, x200). (b) The granulation tissue of the ischemic non-treated group showing negative expression of Pax7 (diaminobenzidine, original magnification, x400). (c) The granulation tissue of the ischemic treated group by BM-MSCs showing cells with positive nuclear staining of Pax3 (diaminobenzidine, original magnification, x200). (d) The granulation tissue of the ischemic treated group by BM-MSCs showing cells with positive nuclear staining of Pax7 (diaminobenzidine, original magnification, x200)

Muscle sections taken from all rats in the 3 groups are immunohistochemically assessed for Pax3 and Pax7 nuclear expression. Muscles of the control group show few scattered satellite cells positive for Pax3 and Pax7. The granulation tissue within the muscles of the ischemic non-treated group shows negative expression of pax3 and pax7 (Figures 5a and 5b). On the other hand, the granulation tissue within the muscles of the ischemic treated group by BM-MSCs shows cells demonstrating nuclear expression of the myogenic marker Pax3 in 5 out of the 7 rats (71%) and Pax7 in 4 out of the 7 rats (57%) (Figures 5c and 5d). This suggests that transplantation of mesenchymal stem cells may become a therapeutic tool to improve functional muscle recovery.

**Table 1:** Genes subjected to amplification and the primer sequences

**Gene **	**Primers**
SDF-1	F: CCAAACTGTGCCCTTCAGAT R: AAGTCCTTTGGGCTGTTGTG
CXCR4	F: ACGGCTGTAGAGCGAGTGTT R: AGGGTTCCTTGTTGGAGTCA
VEGFR2	F: GATGTGGTTCTGAGTCCGTCT R: CATGGCTCTGCTTCTCCTTTG
vWF	F: TAAGTCTGAAGTAGAGGTGG R: AGAGCAGCAGGAGCACTGGT
HIF-1α	F: GTCGGACAGCCTCACCAAACAG R: TAGGTAGTGAGCCACCAGTGTCC
GAPDH:	F: CTCTACTGGCGCTGCCAAGGCT R:GTCCACCACTGCACGTTGG

The MSCs were positive for CD133, CD34, and KDR

**Table 2:** Correlations between different investigated parameters for angiogenesis and endothelial cells differentiation

Parameters	VEGFR2	vWF	CXCR4	SDF-1
VEGFR2	-----	0.984**	0.828**	0.941**
vWF	0.984**	-----	0.755**	0.942**
CXCR4	0.828**	0.755**	-----	0.739**

Values are expressed as correlation coefficients (r)

VEGFR2: Vascular endothelial growth factor receptor 2.vWF: von Willebrand factor. CXCR4: C-X-C chemokine receptor 4. SDF-1: Stromal cell-derived factor-1

** Correlation was significance at *P*≤0.01

## Discussion

Ischemia initiates inflammation as a reaction of necrotic cells followed by reactive oxygen species (ROS) generation [22). Recent pharmacotherapy and surgical approaches are inadequate to entirely restore ischemic tissues and related to a significant adverse effects risk [23). The BM-MSCs secrete bioactive factors for modulation of immunity and angiogenesis (24). BM-MSCs secrete VEGF and differentiate into ECs for prompting angiogenesis in ischemic tissues, and support regeneration and functional recovery of damaged tissues (25). Therefore, BM-MSCs may be a hopeful cell source for ischemic disease therapy. Intramuscular injection into the semimembranosus muscle has been the preferred application in most trials to avoid cell trapping in the pulmonary circulations when transplanted intravenously.

Kiani *et al*. (26) revealed that the difference in CXCR4 expression is interconnected to the hypoxia effects generated through the prompted ischemia. However, CXCR4 expression significantly augmented compared with the basal level, coming in line with the significant increase in its expression in rats with ischemia relative to that in the control group observed in the current study. Additionally, post-treatment of rats with BM-MSCs significantly raised the gene expression level of CXCR4 compared with control and ischemia groups in the current work. These results are consistent with observations of Shiba *et al*. (27) who revealed that stem cells express CXCR4, enhancing angiogenesis in ischemic disease. In the current study, SDF-1 expression significantly increased with BM-MSCs transplantation compared with the control and ischemia groups. This result comes in agreement with the work of Shiota *et al*. (28). 

SDF-1, chemotactic cytokine, shows an essential role in several physiological functions, including angiogenesis and vessel remodeling, and inflammatory responses through interaction with its receptor CXCR4 (29), explaining the positive significant correlation between CXCR4 and SDF-1 in the present work. SDF-1 and CXCR4 facilitate homing, recruitment, and engraftment of preexisting or externally transplanted hematopoietic stem cells in ischemic lesions to repair injuries (30). The mechanism of SDF-1/CXCR4 in facilitating vasculogenesis is chiefly through its synergistic effects with VEGF to facilitate EC proliferation and tube formation by mobilizing, recruiting, and homing CXCR4 bone marrow-derived cells, including endothelial progenitor cells (EPCs) which could differentiate into mature EC, pericyte progenitor cells, smooth muscle progenitor cells, and bone marrow-derived CD45+ vascular modulatory cells which serve as vascular modulators by differentiating into mature EC and smooth muscle cells and by secreting pro-angiogenesis factors. Meanwhile, SDF-1 could prompt EC proliferation and differentiation, and consequently, regulate angiogenesis-related cytokine secretion and exert a synergistic outcome with VEGF on prompting neo-angiogenesis (31). 

Our current work demonstrated that transplantation of BM-MSCs significantly elevated VEGFR2 gene expression levels relative to control and ischemia groups. These data come in line with the observations of other researchers(32) who stated that BM-MSCs developed endothelial phenotype, including VEGFR2 expression. VEGF-A is crucial for EC functions accompanying angiogenesis. In the VEGF system of EC, the utmost prominent ligand-receptor complex (VEGFA/VEGFR2) facilitates the receptor dimerization and specific intracellular tyrosine residue auto-phosphorylation, activating intracellular signal transduction cascades, which promote endothelial cell proliferation, differentiation, migration, survival, permeability, and new vessel formation involved in angiogenesis (33).

In ECs, signaling of VEGFR2 stimulates enormous downstream signaling mediators, involving phosphoinositide-3 kinase (PI3K)/AKT, p38 mitogen-activated protein kinase, and extracellular-signal-regulated kinase-1/2 (ERK-1/2), which serve in a coordinated pattern to start the angiogenic process (10). The PI3K/AKT pathway, through endothelial nitric oxide synthase (eNOS) activation, is responsible for nitric oxide (NO) production, which is a crucial mediator of VEGF-stimulated endothelial permeability, vascular remodeling, and new vessel formation. Additionally, NO is a powerful repressor of nuclear factor-kappa B (NF-κB) contributing to anti-inflammatory effects (34).

Furthermore, *in vivo* differentiation of transplanted BM-MSCs into ECs triggers the production of human endothelial markers such as vWf. This observation comes in agreement with the current data which revealed that gene expression levels of vWF significantly augmented in rats treated with BM-MSCs compared with the control and ischemia groups. *In vivo* study has implicated vWF’s role in vascular inflammation regulation, leukocyte recruitment, vascular permeability, and angiogenesis (35). An intracellular pathway including angiopoietin-2 (Ang-2) storage in Weibel Palade Bodies (WPB) of EC and extracellular pathway has been revealed to affect VEGF/ VEGFR2 signaling, promoting angiogenesis. The extracellular pathway involving vWF binding to αvβ3, a heterodimeric adhesion receptor with several ligands that stabilizes αvβ3 on the surface of the cell and regulates its levels and activity on vascular smooth muscle cells (VSMC) is crucial for their recruitment, supporting maturation of arteries and angiogenesis (36).  

Additionally, vWF interrelates with or adjusts storage of several proteins which have been associated with vascular function and angiogenesis regulation, including interleukin-8, galectin-1and 3, connective tissue growth factor, and insulin-like growth factor binding protein-7 (37). However, a study (38) revealed that VEGF promotes vWF secretion by ECs, via a specific VEGFR2/PLC-γ pathway, confirming the positive significant correlation between VEGFR2 and vWF in the existing study. Interestingly, the significant attenuation in VEGFR2 and vWF levels and the non-significant decrease in the level of SDF-1 gene expression in the ischemia group compared with the control group in the current work may be related to the time and course of ischemia. This observation comes in agreement with that of another study (39) that revealed that VEGFR2 levels were diminished by about 80% matched with ECs from the limb with no ischemia after three days of induced hindlimb ischemia.

A significant high HIF-1α gene expression was registered in the ischemia group and the rats treated with BM-MSCs compared with the control group, but with significant reduction in HIF-1α gene expression in the treated group compared with the ischemic non-treated group. Our observations come in agreement with those of Ramamoorthy and Shi (40), and Palomaki *et al*. (41) who stated that HIF-1α expression is prompted by ischemia and up-regulated in BM-MSCs, respectively. In ischemia, adaptation to hypoxia necessitates numerous gene activations that contribute to angiogenesis, proliferation of cells, energy metabolism, and cell survival. HIF-1 α is a chief transcriptional mediator of the hypoxia reaction and principal regulator of oxygen homeostasis. BM-MSCs are supposed to originate from hypoxic stem cell niches. This assumes that O2 has a crucial role in their regulation. In BM-MSCs, up-regulated HIF-1α is a common regulator for adjusting their metabolic fate and multipotency (41).

Angiogenesis is the process of sprouting new capillaries from pre-existing microvasculature. Angiogenesis is mainly driven by ischemia and up-regulation of ischemia-induced transcription factors like HIF1a, and the genes that are responsive to these transcription factors, such as VEGFa and SDF1. The HIF-1α pathway is a chief regulator of angiogenesis (42). The contribution of HIF-1α in angiogenesis is attributed to transcriptionally triggering numerous angiogenic genes and their receptors such as VEGF/VEGFR2 and erythropoietin, regulating pro-angiogenic chemokines and receptors such as SDF-1 and CXCR4 which triggers the endothelial progenitor cell recruitment to the hypoxia site (43), and augmenting EC division and proliferation by controlling genes included in the cell cycle and replication of DNA (44). However, it is assumed that when the blood flow is adequate to provide oxygen supply to cells, HIF-1α will undergo quick degradation (45), explaining the significant reduction in its gene expression with BM-MSCs treatment compared with the ischemia group in the current study. 

In the current study, results of histopathological examination revealed that muscles from the ischemic non-treated group showed necrosis with dense inflammatory reaction with absent angiogenesis. Muscles from the ischemic treated group by BM-MSCs showed granulation tissue with mild inflammatory reactions, restoration of the normal architecture and the ultrastructure of the muscle, stimulating its regeneration and decreasing the post-ischemic fibrosis by enhancing angiogenesis with new blood vessel formation. The previous finding is in accordance with another study (45). 

Within the PAX transcription factor family, PAX3 and PAX7 play important roles in the different tissues during development. PAX3 regulates neural crest with PAX7. They are also, expressed in parts of the central nervous system. Both factors are key regulators of myogenesis. Pax3 plays a major role during early skeletal muscle formation, while Pax7 predominates during muscle regeneration (46).

In our study, the granulation tissue within the muscles of the ischemic treated group by BM-MSCs shows cells demonstrating nuclear expression of the myogenic marker Pax3 in 5 out of the 7 rats (71%) and Pax7 in 4 out of the 7 rats (57%). This suggests that transplantation of mesenchymal stem cells may become a therapeutic tool to improve functional muscle recovery. In 2008, researchers (47) stated that Pax3 promotes MSCs differentiation towards the myogenic lineage, at the expense of other lineages of mesenchymal tissues including bone, cartilage, and fat and they concluded that there is the potential of regulating transcriptional pathways to direct adult stem cell differentiation. In accordance with our study, another (48) suggests that both transcripts of Pax3 and Pax7 are needed for commitment of BM-MSCs to the myogenic lineage and each transcript has a particular role.

In 2014, researchers (49) tried to clarify the mechanism of how MSC transplantation improves regeneration of injured skeletal muscles. They found that MSCs administration stimulates mobilization, differentiation, and fusion of Pax7-positive satellite cells. The BM-MSCs facilitate regeneration of muscles in Duchenne muscular dystrophy in model mice (50). In a study done in 2019 (51), they found that Pax7 is essential for transcriptional stimulation of myogenic factor 5 (Myf5) in committed myoblasts. A group of researchers (52) demonstrated that genetic ablation of Pax7+ muscle progenitor cells (MPCs, or satellite cells) in a murine model of hindlimb ischemia (HLI) resulted in a complete absence of normal muscle regeneration following ischemic injury, despite a lack of morphological or physiological changes in resting muscle. Compared with the ischemic muscle of control mice (Pax7^WT^), the ischemic limb of Pax7-deficient mice (Pax7^Δ^) was unable to generate significant force 7- or 28-days after HLI. A dramatic increase in adipose infiltration was observed 28 days after HLI in Pax7^Δ^ mice, which replaced functional muscle.

## Conclusion

The most remarkable results in this study confirm the beneficial effects of BM-MSCs on peripheral arterial disease therapy. Transplantation of BM-MSCs has an ameliorating effect on muscle ischemia through promoting angiogenesis confirmed by increased gene expression levels of SDF-1, CXCR4, and VEGFR2, and vWF decreased HIF-1α gene expression. Angiogenesis was detected by new blood vessel formation distal to the obstruction in the gastrocnemius muscle by histopathological examination. Transplantation of BM-MSCs increased myogenic pax3 and Pax7 gene expression in the muscles indicating muscle regeneration.
